# Development of humoral and cellular immunological memory against SARS-CoV-2 despite B cell depleting treatment in multiple sclerosis

**DOI:** 10.1016/j.isci.2021.103078

**Published:** 2021-09-02

**Authors:** Klara Asplund Högelin, Nicolas Ruffin, Elisa Pin, Anna Månberg, Sophia Hober, Guro Gafvelin, Hans Grönlund, Peter Nilsson, Mohsen Khademi, Tomas Olsson, Fredrik Piehl, Faiez Al Nimer

**Affiliations:** 1 Neuroimmunology Unit, Department of Clinical Neuroscience, Karolinska Institutet, Center for Molecular Medicine L8:04, 171 76 Stockholm, Sweden; 2 Division of Affinity Proteomics, Department of Protein Science, KTH Royal Institute of Technology, SciLifeLab, 17165 Stockholm, Sweden; 3 Division of Protein Technology, Department of Protein Science, KTH Royal Institute of Technology, SciLifeLab, 17165 Stockholm, Sweden; 4 Therapeutic Immune Design Unit, Department of Clinical Neuroscience, Karolinska Institutet, Center for Molecular Medicine L8:02, 171 76 Stockholm, Sweden

**Keywords:** Immunology, Virology

## Abstract

B cell depleting therapies (BCDTs) are widely used as immunomodulating agents for autoimmune diseases such as multiple sclerosis. Their possible impact on development of immunity to severe acute respiratory syndrome virus-2 (SARS-CoV-2) has raised concerns with the coronavirus disease 2019 (COVID-19) pandemic. We here evaluated the frequency of COVID-19-like symptoms and determined immunological responses in participants of an observational trial comprising several multiple sclerosis disease modulatory drugs (COMBAT-MS; NCT03193866) and in eleven patients after vaccination, with a focus on BCDT. Almost all seropositive and 17.9% of seronegative patients on BCDT, enriched for a history of COVID-19-like symptoms, developed anti-SARS-CoV-2 T cell memory, and T cells displayed functional similarity to controls producing IFN-γ and TNF. Following vaccination, vaccine-specific humoral memory was impaired, while all patients developed a specific T cell response. These results indicate that BCDTs do not abrogate SARS-CoV-2 cellular memory and provide a possible explanation as to why the majority of patients on BCDTs recover from COVID-19.

## Highlights

• BCDT might blunt antibody responses after COVID-19 infection or vaccination
• Patients with no detectable B cells in the blood might still produce antibodies
• A majority of patients that do not develop antibodies still display a T cell response
• SARS-CoV-2 T-cells produce Th1 cytokines both in patients on BCDT and untreated

## Graphical abstract

**Figure:**
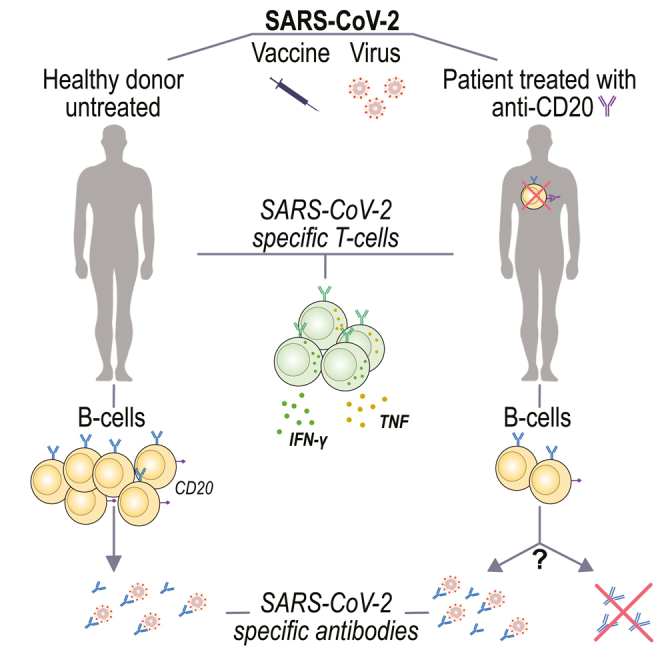

## Introduction

In December 2019, the first cases of infection with a new zoonotic pathogen were detected. Severe acute respiratory syndrome virus-2 (SARS-CoV-2) causes a variety of clinical symptoms and syndromes, ranging from asymptomatic to lethal infections, that are altogether known as coronavirus disease 2019 (COVID-19). The ongoing COVID-19 global pandemic has sparked intense efforts to identify risk factors and characterize the pathophysiology of severe disease courses in order to reduce disease morbidity and mortality while waiting for vaccination. Demographic factors such as advanced age, male sex, and comorbidities such as obesity, chronic obstructive pulmonary disease, and cardiovascular and kidney diseases have all been found to be associated with increased risk for severe disease or mortality (Izcovich et al., 2020).

The immunologic host response to SARS-CoV-2 is complex with different components of the innate and adaptive systems synergistically interacting in the defense against the virus. Importantly, both too little and too much immune activation can be detrimental, as severe immunosuppression as well as cytokine storm syndrome has been implicated as risk factors for severe COVID-19 disease (Lee et al., 2020; Remy et al., 2020; Vabret et al., 2020). Therefore, immunosuppressant therapies for autoimmune diseases on the one hand have been considered risk factors for a more severe disease course, while certain immunomodulators on the other hand have been clinically tested for possible attenuation of hyperinflammatory responses. Such trials have mainly involved modulators of cytokine and chemokine signaling, such as interleukin-6 blockers (Meyerowitz et al., 2020; Stone et al., 2020; Vabret et al., 2020). In parallel, epidemiological studies have assessed if immunosuppressive therapies are associated with more severe COVID-19 (Sadeghinia and Daneshpazhooh, 2020; Schoot et al., 2020).

In particular, B cell depleting anti-CD20 monoclonals, e.g., ocrelizumab (OCR), ofatumumab, and rituximab (RTX), have raised concerns due to their potential to abrogate development of humoral responses to infectious agents including SARS-CoV-2 (Baker et al., 2020; Bar-Or et al., 2020; Houot et al., 2020; Meca-Lallana et al., 2020; Zabalza et al., 2020). B cell depleting therapies (BCDTs) are widely used in hematologic malignancies as well as in a variety of autoimmune diseases, such as rheumatoid arthritis and multiple sclerosis (MS). Emerging epidemiological evidence suggests that anti-CD20 therapies might be associated with a higher risk for a more severe COVID-19 disease course but not increased mortality (Peeters et al., 2020; Safavi et al., 2020; Sormani et al., 2021a, Zabalza et al., 2020). However, caution should be exerted when interpreting these types of data since they rely on spontaneous reporting with difficulties in completely controlling for confounders. In addition, in persons with MS (pwMS), established risk factors seem to outweigh the impact of disease-modulatory therapies (DMTs) on COVID-19 outcomes (Bsteh et al., 2021; Louapre et al., 2020; Zabalza et al., 2020). Nevertheless, a recent pre-pandemic study showed an increased rate of severe infections with B cell depletion compared to other DMTs in pwMS (Luna et al., 2020). Therefore, suggested guidelines have included increased social distancing, choice of another medication than anti-CD20, or extending dosing intervals (Korsukewitz et al., 2020). While epidemiological data to some degree are at hand, it remains unknown if and to what degree individuals with anti-CD20 treatment develop immunity to SARS-CoV-2. The objective of this study was to provide a detailed characterization of humoral and cellular immunity to SARS-CoV-2 in a population-based cohort of patients with relapsing-remitting MS exposed to a number of different DMTs. Our findings suggest that most patients develop cellular immunity to SARS-CoV-2 that does not significantly differ between different DMTs, including anti-CD20-treated patients with suppressed B cell levels.

## Results

### Study participants

**Figure 1:**
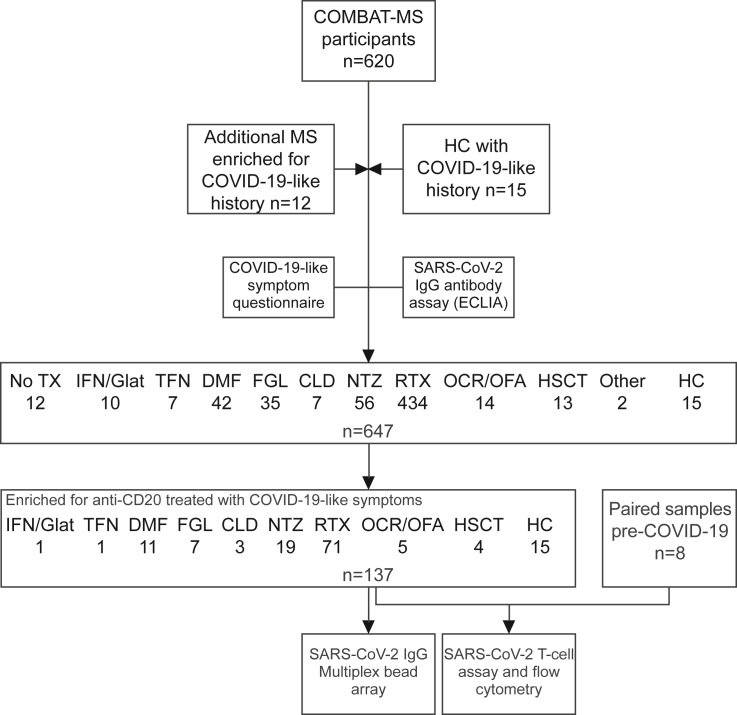
Study design CLD = cladribine; DMF = dimethyl fumarate; ECLIA = electro-chemiluminescence immunoassay; FGL = fingolimod; Glat. = glatirameracetate; HC = healthy controls; HSCT = hematopoietic stem cell transplantation; IFN = interferon-beta; No TX = no treatment; NTZ = natalizumab; OCR = ocrelizumab; OFA = ofatumumab; RTX = rituximab; TFN = teriflunomide.

The study base comprised 620 pwMS participating in the COMBAT-MS, with the addition of 12 pwMS and 15 non-MS controls (healthy controls, HC) enriched for persons that had COVID-19-like symptoms (Figure 1). Eighty percent of HC and 31% of pwMS reported COVID-19-like symptoms after the 1^st^ January 2020, out of which 50% and 4.5% tested positive for SARS-CoV-2 IgG with electro-chemiluminescence immunoassay (ECLIA), respectively. Peripheral blood mononuclear cells (PBMCs) and plasma were collected from all HC and 122 pwMS for further analysis of T cell SARS-CoV-2 reactivity and humoral immunological memory with multiplex bead arrays. PBMCs collected in the pre-COVID-19 period from 8 pwMS participants were used as additional controls in the T cell reactivity assay. The cohort of pwMS treated with RTX was enriched for patients with COVID-19-like symptoms, and therefore, 89% of pwMS on RTX had COVID-19-like symptoms and 18% were seropositive with ECLIA. Tables 1 and S1 show the participant characteristics, and Table S2 shows the number of patients tested with each method.

**Table 1:** Characteristics of the study population

	Study participants	N	Age in years mean ± SD	Female No (%)	EDSS median (min; max)	Patients with COVID-19-like symptoms No (%)	Antibody-positive ECLIA No (%)
Symptom questionnaire SARS-CoV-2 antibodies by electro-chemiluminescence immunoassay	HC	15	43 ± 12.9	10 (67%)	–	12 (80%)	5 (56%)^a^
	MS	632	43 ± 10.3	450 (71%)	2 (0; 7)	196 (31%)	29 (5%)
	No ongoing treatment	12	53.9 ± 9.8	9 (75%)	2.75 (0; 4.5)	3 (25%)	2 (17%)
	Interferon-beta/glatirameracetate	9/1	52.2 ± 8.9	9 (90%)	2.25 (1; 4)	3 (30%)	1 (10%)
	Teriflunomide	7	48.3 ± 7.3	5 (71%)	3.5 (0; 6.5)	4 (57%)	0 (0%)
	Dimethyl fumarate	42	42.5 ± 9.1	26 (62%)	1.25 (0; 4.5)	12 (29%)	2 (5%)
	Fingolimod	35	44.8 ± 9.6	25 (71%)	2 (0; 7)	11 (31%)	1 (3%)
	Cladribine	7	40.6 ± 4.4	4 (57%)	2.5 (1; 4.5)	3 (43%)	1 (14%)
	Natalizumab	56	38.9 ± 7.7	46 (82%)	1.5 (0; 6)	28 (50%)	1 (2%)
	Rituximab	434	43 ± 10.6	313 (72%)	2 (0; 7)	122 (28%)	19 (4%)
	Ocrelizumab/Ofatumumab	13/1	39.3 ± 10	8 (57%)	1.5 (0; 3.5)	6 (43%)	1 (7%)
	HSCT	13	38.5 ± 6.1	3 (23%)	4 (1.5; 6)	3 (23%)	1 (8%)
	Other	2	47 ± 4.2	2 (100%)	4.5 (3; 6)	0 (0%)	0 (0%)
SARS-CoV-2 antibodies by multiplex bead array T cell assays	MS	122	43 ± 9.8	89 (73%)	2 (0; 6.5)	92 (75%)	20 (16%)
	Interferon-beta	1	62	0 (0%)	2	1 (100%)	1 (100%)
	Teriflunomide	1	55	0 (0%)	4	0 (0%)	0 (0%)
	Dimethyl fumarate	11	41.9 ± 12	8 (73%)	1 (0; 4.5)	7 (64%)	2 (18%)
	Fingolimod	7	49.1 ± 10.5	6 (86%)	2 (1; 6.5)	4 (57%)	1 (14%)
	Cladribine	3	40 ± 3.6	1 (33%)	2.5 (1.5; 2.5)	1 (33%)	1 (33%)
	Natalizumab	19	38.9 ± 6.2	15 (79%)	1.5 (0; 3.5)	9 (47%)	1 (5%)
	Rituximab	71	43.7 ± 10.1	55 (77%)	2 (0; 6)	63 (89%)	13 (18%)
	Ocrelizumab	5	42.4 ± 12.7	2 (40%)	1.5 (0; 2)	5 (100%)	1 (20%)
	HSCT	4	42.8 ± 4.9	1 (25%)	2.75 (1.5; 6)	2 (50%)	0 (0%)

EDSS = expanded disability status scale; ECLIA = electroluminescence immunoassay; HC = healthy controls; MS = multiple sclerosis; HSCT = hematopoietic stem cell transplantation.

a Six HC were not tested with ECLIA.

### Patients with MS treated with immunosuppressants develop SARS-CoV-2 humoral and cellular immunological memory

**Figure 2:**
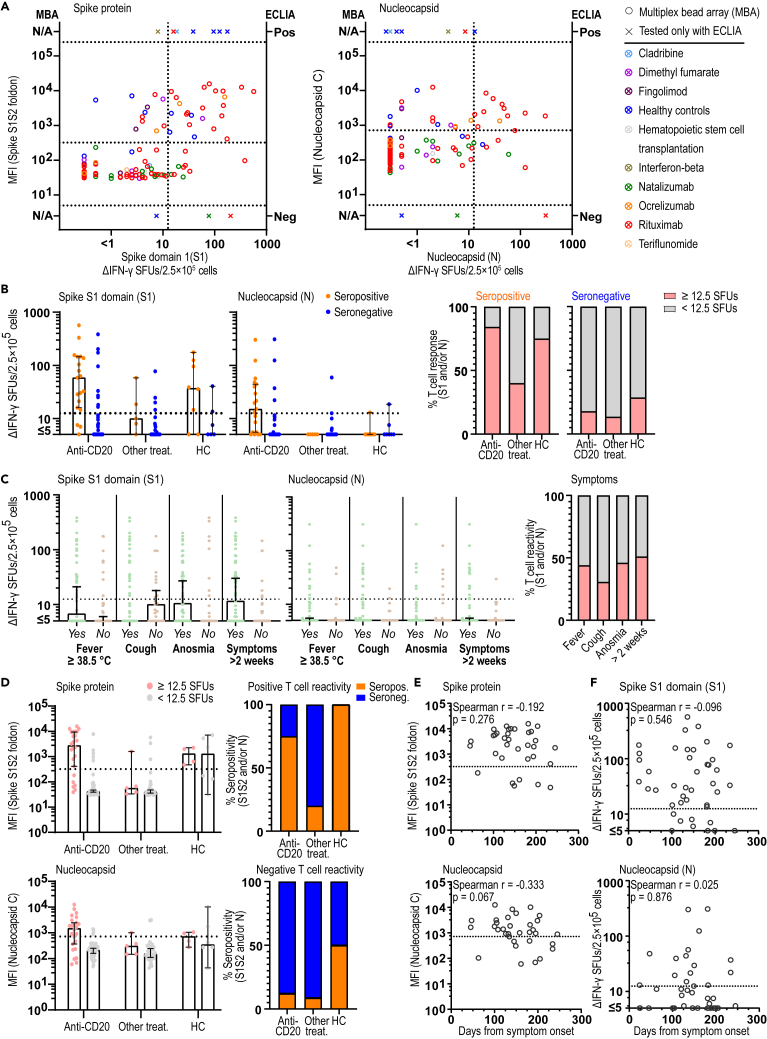
Patients with MS treated with immunosuppressants develop SARS-CoV-2 humoral and cellular immunological memory (A) Correlation of SARS-CoV-2 antibody levels as measured by MFI of S1S2 or N with ΔIFN-γ SFUs after stimulation with S1 (n = 122) or N (n = 122) peptides, respectively. Ten samples that were only tested with ECLIA are also shown. (B) Number of ΔIFN-γ SFUs after stimulation with S1 (n = 132) or N peptides (n = 132) in seropositive or seronegative patients on anti-CD20 (n = 75) or other immunosuppressive treatment (n = 42) and in HC (n = 15). The percentage of persons with SARS-CoV-2-positive T cell immunity per serostatus and treatment group or HC. (C) Number of ΔIFN-γ SFUs after stimulation with S1 or N peptides in patients (n = 88) and HC (n = 11) with specific COVID-19-like symptoms. The percentage of SARS-CoV-2-positive T cell immunity per specific COVID-19-like symptom. (D) Anti-SARS-CoV-2 antibody MFI for S1S2 and N in persons with positive or negative T cell immunity and on anti-CD20 (n = 73) or other immunosuppressive treatment (n = 39) and in HC (n = 10). The percentage of positive and negative serology status in pwMS and HC with positive or negative SARS-CoV-2 T cell immunity is shown. (E and F) Correlation of MFI (n = 34) or ΔIFN-γ SFU (n = 42) measured with days between sampling and COVID-19-like symptoms. Spearman r and p values are shown. Dots represent individual data points while X represents samples that were only tested with ECLIA. Box plots represent median and 95% CI. See also Tables S1, S2 and Figure S1. ECLIA = electro-chemiluminescence immunoassay; HC = healthy controls; MFI = median fluorescent intensity; pwMS = persons with multiple sclerosis; SFU = spot forming unit.

T cell reactivity against SARS-CoV-2 in sampled patients and controls was assessed by measuring IFN-γ spot forming units (ΔSFU) in FluoroSpot assay using peptides for spike protein (S), N-terminal spike domain (S1), membrane glycoprotein domain (M), and nucleocapsid (N) of SARS-CoV-2. Results from 6 paired samples collected before and after the pandemic confirmed specific T cell reactivity to the SARS-CoV-2 peptides S, S1, and N exclusively in the post-pandemic seropositive samples (Figure S1A). In addition, a high proportion of seronegative samples displayed high ΔIFN-γ SFU in FluoroSpot for the M and S peptides, suggesting them to be less specific for SARS-CoV-2 and were thus excluded from the analyses (Figure S1B) (Sekine et al., 2020). Importantly, both levels of antibodies measured with the multiplex bead array and IFN-γ responses in FluoroSpot correlated well between SARS-CoV-2 spike and nucleocapsid domains in each method, respectively, while instead there was no evident correlation between the strength of humoral and T cell immunity (Figures S1C and S1D). Based on this and previous studies, we defined SARS-CoV-2 T cell positive samples as those that had ≥12.5 ΔIFN-γ SFU/2.5 × 10^5^ cells for S1 and/or N (Sekine et al., 2020). Eighteen out of 24 pwMS and 6 out of 8 HC that were SARS-CoV-2 seropositive with either ECLIA or multiplex bead array for spike (S1S2) and nucleocapsid also displayed SARS-CoV-2 T cell reactivity for S1 or N peptides in FluoroSpot (Figure 2A). Seven pwMS were positive in the multiplex bead array for only one of the two peptide domains (spike S1S2 foldon or nucleocapsid C) and hence were considered as seronegative in the final assessment. Of those seven, three displayed SARS-CoV-2 T cell reactivity for S1 or N peptides in the FluoroSpot assay. Four patients with T cell reactivity were negative with ECLIA but tested seropositive with the multiplex bead array, while no individual displayed positivity with ECLIA and was seronegative with the multiplex bead array. These four patients displayed a lower MFI for nucleocapsid C (p = 0.02) but otherwise did not differ in other factors including time from infection (p = 0.13) compared to those being positive in both assays. Of note, only 3 out of 19 (16%) of seropositive anti-CD20-treated pwMS lacked detectable T cell responses (Figure 2B). Although this could depend on the few samples tested, it may also be affected by the HLA type of these patients and/or the limited number of SARS-CoV-2 peptides tested (Nguyen et al., 2020). On the contrary, 16.4% (9/55), 13.5% (5/37), and 28.6% (2/7) of pwMS treated with anti-CD20, other DMT, and HC, respectively, displayed T cell reactivity while being negative for antibodies. This may relate to loss of previously existing humoral response, as previously reported (Anna et al., 2020; Perreault et al., 2020), but in case of pwMS, it may also relate to the effects of DMTs (Figures 2A and 2B). In general, the strength of memory T cell response did not noticeably differ between seropositive and seronegative/T cell-positive samples and was at least as good in the anti-CD20-treated group (RTX and OCR) as in patients with other treatments and HC (Figure 2B).

There was a tendency that donors with fever, anosmia, and symptoms more than 2 weeks display higher frequencies of SARS-CoV-2 memory T cell responses as compared to donors only reporting cough, suggesting a lower specificity of the latter for COVID-19 (Figure 2C). There was, however, no apparent difference in the levels of anti-SARS-CoV-2 antibodies between treatment types (Figure 2D) or reported symptoms (data not shown). A tendency for a decrease over time in antibody levels to N (r = −0.333, p = 0.067), but not to S, or T cell memory responses to S1 or N was observed, partly explaining the observation that antibodies and T cell reactivity did not correlate (Figures 2E and 2F). No apparent effect of age or sex was seen (data not shown). Unfortunately, few subjects had been tested with PCR, since in Sweden such testing during the first phase of the pandemic was mainly restricted to hospital admissions (Table S1). However, all 7 out of 71 pwMS on RTX treatment with a positive SARS-CoV-2 PCR test also had antibodies and/or T cell immunological memory, while all 8 pwMS that reported negative PCR did not.

These findings indicate that cellular and humoral immune response against SARS-CoV-2 may arise or persist independently and that BCDTs do not prevent development of SARS-CoV-2 immunity.

### SARS-CoV-2-specific T cells from patients with MS on anti-CD20 treatment are functional

**Figure 3:**
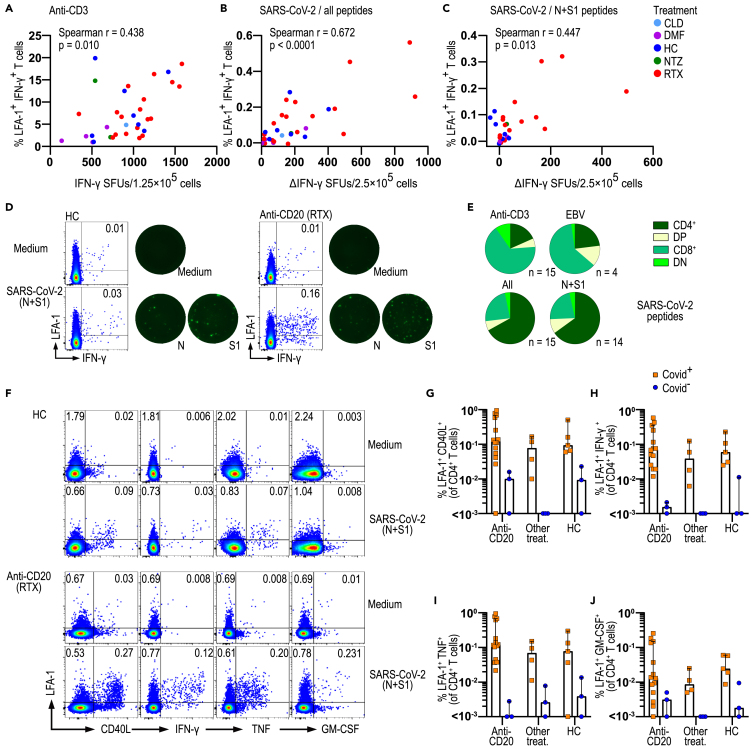
SARS-CoV-2-specific T cells from patients with MS on anti-CD20 treatment are functional (A–C) Correlation of ΔIFN-γ SFUs measured by FluoroSpot with percentages of LFA-1^+^ IFN-γ^+^ T cells stimulated with anti-CD3 (n = 34) or four SARS-CoV-2 peptides (N, S, S1, and M, n = 34) or only N and S1 SARS-CoV-2 peptides in pwMS and HC (n = 30). (D) Representative dotplots of LFA-1 and IFN-γ expression of total CD3^+^ T cells with corresponding IFN-γ FluoroSpot image of one HC and one pwMS under anti-CD20 treatment. (E) Repartition of LFA-1^+^ IFN-γ^+^ specific T cells between CD4 and CD8 among pwMS with anti-CD20 treatment following stimulation as in (A–C) (n = 14 or 15) and with EBV peptides (n = 4). (F) Representative dotplots of CD4^+^ T cell expression of LFA-1 with CD40L, IFN-γ, TNF, and GM-CSF from one HC and one pwMS with anti-CD20 treatment following a culture with medium or in the presence of SARS-CoV-2 peptides N and S1. (G–J) Percentages of LFA-1^+^CD40L^+^, LFA-1^+^IFN-γ^+^, LFA-1^+^TNF^+^, and LFA-1^+^GM-CSF^+^ among CD4^+^ T cells in HC (n = 8) and pwMS with anti-CD20 treatment (n = 18) or with other therapies (n = 7). Spearman r and p values are shown. Dots represent individual data points. Box plots represent median and 95% CI. See also Figure S2. CLD = cladribine; DMF = dimethyl fumarate; DN = double-negative; DP = double-positive; EBV = Epstein-Barr virus; HC = healthy controls; MFI = median fluorescent intensity; NTZ = natalizumab; pwMS = persons with multiple sclerosis; RTX = rituximab; SFU = spot forming unit.

Next, we further assessed the cellular immunological memory of COVID-19-positive patients, i.e., patients that were positive for SARS-CoV-2 in at least one of the assays. PBMCs were stimulated with either anti-CD3 as a positive control, only medium as a negative control, Epstein-Barr virus (EBV) peptides as another virus control, and a mixture of peptides of all four domains (N, S, S1, and M) or only the two SARS-CoV-2 domain peptides (S1, N) that are specific for SARS-CoV-2. To determine which cells produce IFN-γ following stimulation, we analyzed the change of LFA-1 conformation in response to T cell activation in combination with intracellular cytokine staining using flow cytometry (Dimitrov et al., 2018; Schollhorn et al., 2021). The observation that the percentage of LFA-1^+^IFN-γ^+^ T cells correlated well with FluoroSpot IFN-γ SFU for anti-CD3 (r = 0.438, p = 0.01), N, S, S1, and M (r = 0.672, p < 0.0001) and N and S1 (r = 0.447, p = 0.013) peptide stimulation (Figures 3A–3D) confirmed the specificity of this approach. Analyses of LFA-1^+^IFN-γ^+^ T cells revealed that nearly two-third of activated T cells were CD4^+^ for SARS-CoV-2 peptides and approximately 15% were CD8^+^. Notably, these proportions were reversed following anti-CD3 and EBV peptide stimulation, with a vast majority of activated T cells being CD8^+^ (Figure 3E). Further investigation is necessary to establish if the observed CD4^+^ T cell skewing with SARS-CoV-2 peptides is a consequence of peptide length or if it reflects the biological characteristics of the response to this virus. Importantly, the co-expression of LFA-1 with either CD40L, IFN-γ, TNF, or GM-CSF in CD4^+^ and CD8^+^ T cells was similar in pwMS on anti-CD20 treatment and in HC and was at least as high in patients on anti-CD20 treatment as in HC and pwMS on other treatments (Figures 3F–3J and S2). Of note, the highest T cell responses were observed in 2 patients that had been hospitalized, in accordance with the reported association between immune response and symptoms (Sekine et al., 2020). Our results thus point to CD4^+^ T cells as IFN-γ producing cells upon SARS-CoV-2 peptide stimulation in this FluoroSpot setting. Importantly, the T cell response of pwMS following SARS-CoV-2 is functional and similar to that of healthy controls, even after BCDT.

### Circulating follicular T helper cells correlate with SARS-CoV-2 antibody levels

**Figure 4:**
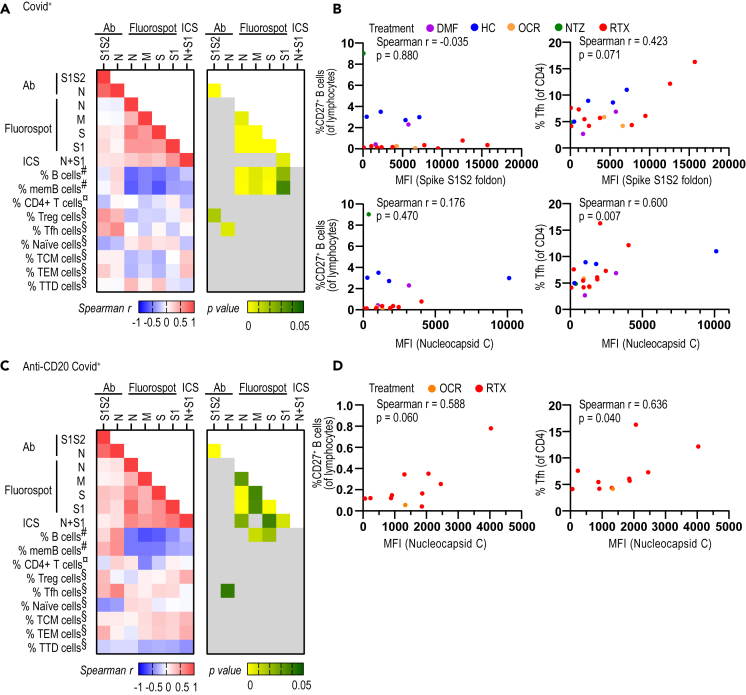
Circulating follicular T helper cells correlate with SARS-CoV-2 antibody levels. PwMS and HC that were SARS-CoV-2 positive in either serological and/or T cell assays are included (A) Correlation matrix representing Spearman r (left panel) and the corresponding p values (right panel) between the COVID-19-specific antibody levels (n = 40), ΔIFN-γ levels measured by FluoroSpot (n = 50), and percentages of LFA-1^+^IFN-γ^+^ T cells (n = 14), with the percentage of immune cells determined by FACS in COVID-19-positive pwMS and HC. (B) Correlation between percentages of memory CD27^+^ B cells (left images) and Tfh cells (right images) with COVID-19-specific antibody levels (MFI) for spike S1S2 (upper images) and nucleocapsid C (lower images). (C) Correlation matrix representing Spearman r and their corresponding p value between the COVID-19-specific antibody levels immune response (n = 26), IFN-γ levels measured by FluoroSpot (n = 28) and percentages of LFA-1^+^IFN-γ^+^ T-cells (n = 13), with the percentage of immune cells determined by FACS in Covid^+^ (n = 13) pwMS with anti-CD20 treatment. (D) Correlation between percentages of Tfh cells with COVID-19-specific antibody levels for nucleocapsid C in COVID-19-positive pwMS with anti-CD20 treatment (n = 11). DMF = dimethyl fumarate; HC = healthy controls; ICS = intracellular staining; memB = memory B; MFI = median fluorescence intensity; NTZ = natalizumab; OCR = ocrelizumab; pwMS = persons with multiple sclerosis; RTX = rituximab; TCM cells = central memory T cells; TEM cells = effector memory T cells; Tfh = T follicular helper cells; Treg = T regulatory; TTD cells = terminally differentiated T cells.

We then investigated which B and T cell subpopulations were associated with humoral and cellular immunological memory toward SARS-CoV-2, respectively. As described previously, antibody levels for S1S2 and N correlated well with each other but did not correlate to the strength of the T cell memory response (Figures 4A and S1C and S1D). The IFN-γ response correlated well between the two SARS-CoV-2-specific peptide domains (N, S1) and with LFA-1^+^IFN-γ^+^, further confirming the utility of this technique to assess antigen-specific T cell responses. The percentages of B and memory B lymphocytes displayed an inverse correlation with the strength of T cell memory when checked in all COVID-19-positive patients. This possibly reflects the fact that patients on anti-CD20 treatment with low B cells develop a robust cellular immunological memory to SARS-CoV-2 (Figures 4A and 4B). The percentage of T regulatory cells correlated with levels of S1S2 antibodies, perhaps indicating that both are affected by the severity of COVID-19 (Figure 4A) (Wang et al., 2020). Circulating T follicular helper cells (Tfh) reflect the Tfh activity in lymph nodes that help antigen-specific B cells to produce antibodies, which has been found to correlate with SARS-CoV-2 antibody levels (Crotty, 2019; Juno et al., 2020). Accordingly, we here found a correlation between circulating Tfh cells and nucleocapsid C antibody levels, both in the whole COVID-19-positive cohort (Figures 4A and 4B) and when separately analyzing the COVID-19-positive anti-CD20-treated patient group (Figures 4C and 4D). We therefore confirm the association between Tfh cells and antibody responses.

### Immunological memory to SARS-CoV-2 is evident in RTX-treated pwMS irrespective of B cell depletion status

**Figure 5:**
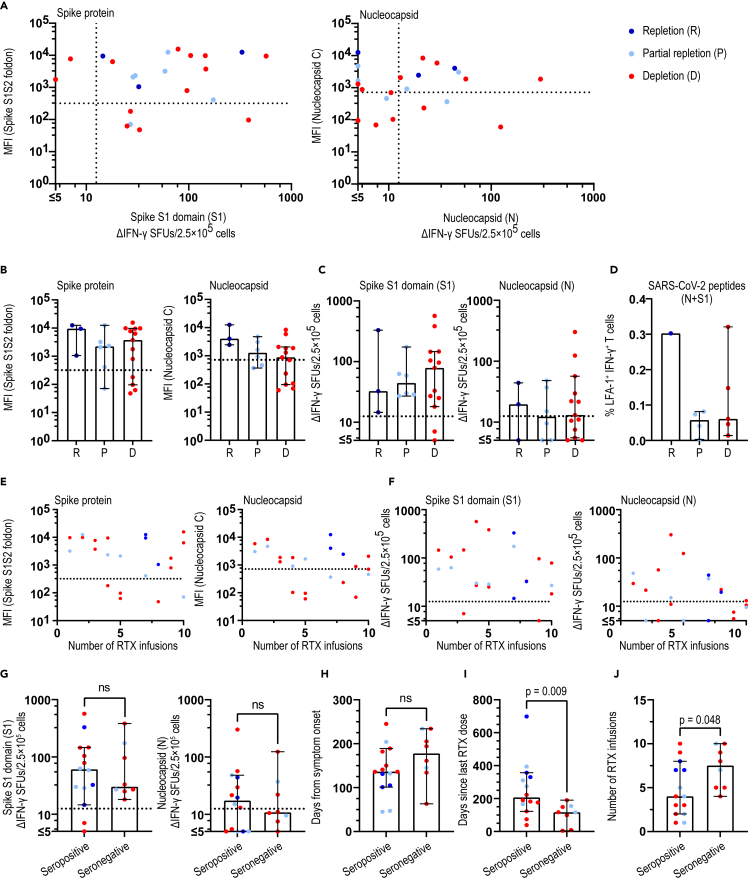
Immunological memory to SARS-CoV-2 is evident in RTX-treated pwMS irrespective of B cell depletion status. Patients that were SARS-CoV-2 positive in either serological and/or T cell assays are included (A) Correlation of SARS-CoV-2 antibody levels for S1S2 or N with ΔIFN-γ SFUs for S1 (n = 22) or N (n = 22) peptides, respectively. (B and C) Anti-SARS-CoV-2 antibody levels for S1S2 and N and number of ΔIFN-γ SFUs for S1 or N peptides in patients with B cell repletion, partial repletion, and depletion. No significant differences were seen. (D) Percentages of LFA-1^+^IFN-γ^+^ in patients with B cell repletion (n = 1), partial repletion (n = 4), and depletion (n = 5). (E) Correlation of antibody levels for S1S2 and N with number of RTX infusions. (F) Correlation of ΔIFN-γ SFUs for S1 and N peptides with number of RTX infusions. (G–J) Comparison of seropositive (n = 14) and seronegative (n = 8) patients regarding number of ΔIFN-γ SFUs for S1 (p = 0.803) or N (p = 0.815) or days from symptom onset to sampling (p = 0.140) or days from last RTX dose to symptom onset (p = 0.009) or number of RTX infusions (p = 0.048). (A–C, E–J) B cell depletion status; n = 3 repletion, n = 6 partial repletion, n = 13 depletion. Dots represent individual data points. Box plots represent median and 95% CI. Mann-Whitney test was used for statistical analysis and p values ≤ 0.05 were considered significant. See also Table S3 and Figure S3. MFI = median fluorescent intensity; ns = non-significant; pwMS = persons with multiple sclerosis; RTX = rituximab; SFU = spot forming unit.

To better understand how RTX affects humoral and cellular immunological memory after COVID-19, we stratified anti-CD20-treated patients into those that at the time of COVID-19-like symptom were (a) completely B cell depleted (<0.01; B cell count × 10^9^/L), (b) partially B cell repleted (0.01–0.08; B cell count × 10^9^/L), and (c) completely B cell repleted (>0.08; B cell count × 10^9^/L). Among the 26 COVID-19-positive patients, 7 out of 14 with complete B cell depletion status, 5/7 with partial repletion, and 3/3 with complete repletion status developed antibodies. Two patients with complete depletion were seropositive with the multiplex bead array but not with ECLIA and displayed more than 5 but less than 11 IFN-γ^+^ spots after stimulation with SARS-CoV-2 peptides (Figures 5A and 5B; Table S3). All but 2 patients with complete depletion displayed SARS-CoV-2 T cell memory without any apparent effect of depletion status on the strength of T cell reactivity measured by FluoroSpot or on SARS-CoV-2 T cell memory functionality measured by LFA-1 and intracellular cytokine expression, respectively (Figures 5C and 5D). In addition, one patient with complete depletion and one patient with partial depletion not being tested with the multiplex bead array displayed T cell memory (data not shown).

Recent epidemiological studies have indicated a reduced ability of the immune system of pwMS on anti-CD20 to produce antibodies (Sormani et al., 2021b; Zabalza et al., 2020). We therefore investigated what factors might affect the SARS-CoV-2 antibody production in pwMS on RTX treatment. The number of previous RTX infusions did not correlate with SARS-CoV-2 antibody levels or the strength of T cell memory (Figures 5E and 5F). When separating seropositive and seronegative patients, they did not differ in regards to strength of T cell reactivity. However, seronegative pwMS displayed less time from last RTX dose to COVID-19 symptoms (p = 0.009) and higher number of previous RTX doses (p = 0.048) (Figures 5G–5J). We cannot exclude a partial effect of waning of antibodies in seronegative pwMS since we saw a correlation (p = 0.029) between time from last RTX dose to symptoms and time from symptoms to sampling (Figure S3), and thus, those effects were lost in multivariate analyses (data not shown). Of note, two patients that developed COVID-19 within 2 weeks of last RTX infusion remained seronegative, while displaying a robust memory T cell response. While short time since RTX treatment potentially impairs B cell responses, the lack of SARS-CoV-2 antibody response may also be the result of waning antibody titers, which were tested 205 and 234 days after COVID-19, respectively (Figure S3) (Ibarrondo et al., 2020). Interestingly, a patient that had received a RTX infusion more than two years ago while still being completely depleted on repeated testing did not develop antibodies but displayed T cell reactivity to SARS-CoV-2.

These results demonstrate that absence of measurable B cell levels in peripheral blood does not prevent development of humoral response and development and strength of T cell response against SARS-CoV-2.

### Immunological memory and functionality of cellular immunological memory in pwMS on other immunosuppressive/immunomodulatory treatments

Although the number of pwMS treated with DMTs other than RTX was low, we nevertheless investigated how these affected humoral and cellular SARS-CoV-2 immune responses in order to provide a preliminary indication and a basis to compare with RTX. Three pwMS on OCR (days since last infusion; 202, 214, 222), another anti-CD20 therapy, tested seropositive with either ECLIA and/or multiplex bead array, suggesting that OCR, as for RTX, does not prevent SARS-CoV-2-specific antibody production. One pwMS on interferon-beta, two pwMS on dimethyl fumarate, a treatment that increases oxidative burst and dampening of MS-associated adaptive immune responses (Carlstrom et al., 2019; Luckel et al., 2019), two patients on fingolimod, a drug that blocks the egress of CCR7^+^ naive and central memory T cells from the lymph nodes (Pappu et al., 2007), one pwMS on cladribine, a drug that depletes B and T cells, and one pwMS on natalizumab, a VLA-4 blocker that impedes the transmigration of lymphocytes into the brain (Piehl, 2020), tested positive in ECLIA and/or multiplex bead array suggesting that these immunotherapies used in pwMS do not impede a humoral anti-viral immune response against SARS-CoV-2. The IFN-γ T cell memory response after stimulation with the SARS-CoV-2 domain peptides is shown in Figure S4. Although treatment-induced lymphopenia does not abrogate the development of T cell immunity, 3 out of 5 seropositive pwMS treated with other DMTs displayed low or undetectable SARS-CoV-2-specific T cell response in both assays. Further investigations are needed to corroborate these observations in larger cohorts.

### Specific T cell response after SARS-CoV-2 vaccination in patients treated with anti-CD20

**Figure 6:**
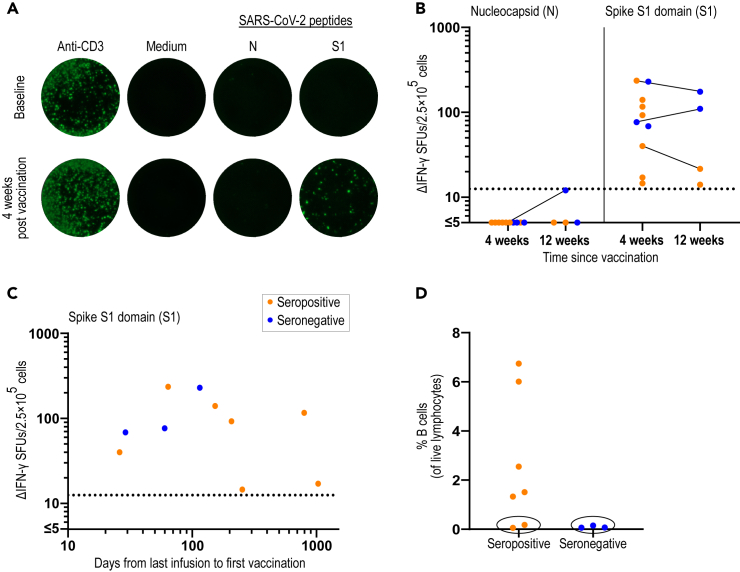
Development of T cellular immune response to the spike protein of SARS-CoV-2 in anti-CD20-treated pwMS after vaccination (A) Images of the IFN-γ FluoroSpot after stimulation with the positive control (anti-CD3), negative control (medium only), and SARS-CoV-2 specific peptides (N and S1) in one pwMS on anti-CD20 before and 4 weeks after SARS-CoV-2 vaccination. (B) Serology status and number of ΔIFN-γ SFUs after stimulation with N or S1 peptides four (n = 10) and twelve (n = 4) weeks after SARS-CoV-2 vaccination in pwMS on anti-CD20. (C) Serology status and number of ΔIFN-γ SFUs after stimulation with S1 peptides 4 weeks after vaccination (n = 10) and days from last anti-CD20 infusion to first vaccine dose. (D) Percentages of B cells in seropositive (n = 7) and seronegative (n = 3) pwMS 4 weeks after vaccination. Samples with <0.2% B cells of live lymphocytes are marked with a black circle. pwMS = persons with multiple sclerosis; SFU = spot forming unit.

To further examine the effect of BCDT on SARS-CoV-2 immunity, the humoral and cellular immune responses were determined in pwMS 4 to 12 weeks after the second dose of SARS-CoV-2 mRNA vaccine. The T cell response was analyzed both before and 4 weeks after vaccination for one patient and was negative for both the N and S1 peptides at baseline but became positive only for S1 after vaccination (Figure 6A). After 4 weeks, 7 out of 10 (70%) patients had antibodies to the spike protein and 10 out of 10 (100%) had a positive T cell response exclusively to the S1 peptides. After 12 weeks, one patient had seroconverted from positive to negative but all patients (3 out of 3) still had a specific T cell response (Figure 6B). To be noted, for the seroconverted patient, the 12-week sample was analyzed using a different antibody detection method with a higher cutoff level for seropositivity. To examine which factors that may influence the development of humoral immunity in anti-CD20-treated pwMS, the time from last infusion to first vaccine dose for each patient with MS was evaluated. Interestingly, for the patients who had received an infusion within 4 months before their first dose, only two out of five (40%) had detectable SARS-CoV-2-specific antibodies 4 weeks after vaccination. The remaining patients with more than 4 months since last infusion, all five out of five (100%) had detectable antibodies 4 weeks after vaccination. However, regardless of time since last infusion or serology status, all pwMS developed a robust and specific T cell response (Figure 6C). When analyzing the percentages of B cells, all seronegative samples displayed less than 0.2% B cells of live lymphocytes. Interestingly, among the seropositive pwMS, there were also two samples with less than 0.2% B cells (Figure 6D), indicating that a virtual absence of B cells in peripheral blood is not a good predictor of not producing detectable antibodies after vaccination. These data also support the observation that SARS-CoV-2-specific T cells can arise and persist even in the absence of detectable SARS-CoV-2 antibodies in pwMS that are being treated with anti-CD20.

## Discussion

We here determined humoral and cellular immune responses to SARS-CoV-2 in a large population-based cohort of pwMS being treated with different DMTs. Since the start of the COVID-19 pandemic, concerns have been raised regarding a worsened COVID-19 disease course, increased risk of re-infection, and dampened vaccine responses in patients treated with immunomodulators, in particular anti-CD20 therapies. Indeed, pwMS treated with anti-CD20 display increased susceptibility to severe infections compared to those treated with interferons, even if this increased risk mainly regards bacterial rather than viral infections (Luna et al., 2020; Montalban et al., 2017). Our study did not aim to provide data on a possible clinical impact of DMT on susceptibility to COVID-19 or its resulting severity. However, in our population-based COMBAT-MS cohort of 620 patients, out of which at least 4.5% had had COVID-19 as verified with ECLIA, 2 patients (both on rituximab) required hospitalization, arguing against a major clinical impact in this cohort.

In order to extend these epidemiological observations to an immunological context, also prompted by the ongoing pandemic, we determined immunological responses to SARS-CoV-2 in a population-based cohort of patients with MS participating in the ongoing prospective COMBAT-MS study. Interestingly, we find that the immune system of all patients except two treated with RTX reacts to acute COVID-19 infection by producing a functional T cell response irrespective of B cell depletion status. The two patients with no T cell response displayed antibodies, which may relate to the combination of HLA type and the peptides used in this study rather than being biological. Thus, we did not observe any apparent effect of B cell depletion status, number of previous RTX infusions, and time between RTX infusion and COVID-19 symptoms regarding the development, strength, and functionality of the cellular response. Half of the patients with complete B cell depletion, as reflected in the blood, that displayed a T cell response displayed also humoral immunological memory. While circulating B cells are greatly impacted by RTX treatment and those are reflected by regular blood tests, there are reports of residual B cell populations in other body compartments, such as bone marrow and lymphoid follicular structures (Nakou et al., 2009; Ramwadhdoebe et al., 2019), which could explain why a humoral immune response can develop in the context of apparent complete B cell depletion. We saw in accordance with previous studies (Bar-Or et al., 2020; Louapre et al., 2021; Zabalza et al., 2020) that time from last RTX dose and number of infusions might affect the humoral response, while not curbing development of a robust cellular memory.

Our results further suggest that lymphopenia induced by other MS DMTs in general does not noticeably affect the quantitative or qualitative aspects of the resulting SARS-CoV-2 adaptive immune response, even though SARS-CoV-2-induced lymphopenia has been shown to correlate with severe COVID-19 (Weiskopf et al., 2020). Indeed, two lymphopenic pwMS, one on dimethyl fumarate and one on cladribine developed both antibodies and T cell response against SARS-CoV-2. It is currently unclear to what degree the cellular and humoral immune responses contribute together or separately toward long-lasting protection against re-infection, which needs to be addressed in further studies (Jarjour et al., 2021).

Notably, we here studied a low dose RTX (500 mg) protocol, which is lower than the approved dose for rheumatoid arthritis, and is based on the notion that memory B cells rather than B cells in general are implicated in MS immunopathogenesis and which also translates in a long-lasting effect after interrupting anti-CD20 therapy (Baker et al., 2017; Jelcic et al., 2018; Juto et al., 2020; Novi et al., 2020). In addition, dosing intervals had been extended already before the start of the pandemic, explaining why a proportion of patients had dosing intervals in the range of 9–24 months or more. However, the observations that cellular immunological responses were not affected by depletion status or time since last dose and that pwMS on the more potent B cell depleting OCR dosing regimen also displayed an adaptive response suggest that the results of the current study can be extrapolated to other doses of RTX and other anti-CD20 therapies (Evertsson et al., 2020). A further finding of our study is that the proportion of pwMS that tested positive for SARS-CoV-2 in the routine antibody test (ECLIA; 4.5%) is lower than the 11.6% seropositive prevalence reported in healthy blood donors by the Public Health Agency of Sweden for the Stockholm population already in June 2020 (https://www.folkhalsomyndigheten.se/contentassets/376f9021a4c84da08de18ac597284f0c/pavisning-antikroppar-genomgangen-COVID-19-blodgivare-delrapport-2.pdf). We believe that this difference can be explained by a higher degree of precaution exerted by pwMS, geographical heterogeneity within Stockholm in the spread of the pandemic, and a possible waning in the humoral response with time since infection since samples in this study were collected mainly in the third quarter of the year (Ibarrondo et al., 2020; Perreault et al., 2020).

In a small cohort of pwMS on anti-CD20 with no previous COVID-19-like history that received anti-SARS-CoV-2 mRNA vaccination, we observed a similar type of development of immunological memory as in the postinfectious group of pwMS i.e. that development of humoral memory might be impaired, depending mainly on time since last infusion and B cell depletion status, but that cellular immunity develops and persists independently. More importantly, they also indicate that although the presence of B cells is important for development of anti-SARS-CoV-2 antibodies, B cells are not the main players involved in antigen presentation for the development of a T cell response following mRNA vaccination since all eleven patients in our study developed a T cell response.

In summary, we here demonstrate that pwMS on anti-CD20 treatment develop an immunological SARS-CoV-2 T cell memory response that displays similar characteristics as pwMS on other DMTs and HC, while the humoral response is affected by treatment including a tendency of decreasing antibody levels but with a remaining strong T cell response (Ibarrondo et al., 2020; Maillart et al., 2020; Peng et al., 2020; Wallach and Picone, 2021; Weiskopf et al., 2020). Furthermore, the post-vaccination data are in accordance with previous studies reporting an attenuated antibody response to SARS-CoV-2 mRNA and also other types of vaccines for patients on anti-CD20 treatment (Achiron et al., 2021; Baker et al., 2020; Bar-Or et al., 2020; Bigaut et al., 2021; Gallo et al., 2021; Guerrieri et al., 2021) and also demonstrate that absence of B cells in blood is a poor predictor of capacity to develop a humoral response. In combination with recent literature that demonstrates a contribution of T cell immunity in COVID-19 (McMahan et al., 2021; Soresina et al., 2020), even in the absence of detectable antibodies, they help explain why most of the patients on anti-CD20 treatment recover and suggest a potential effect of vaccination even in the absence of B cells.

### Limitations of the study

A limitation of this study is that most individuals were not confirmed with PCR testing for SARS-CoV-2 during the acute phase due to the lack of testing capacity early in the pandemic. Thus, since patients on anti-CD20 treatment are generally more prone to respiratory tract infections (Luna et al., 2020), it is likely that early in the pandemic and while PCR verification of patients was lacking, symptoms such as cough and fever might have been caused by other pathogens but still mistaken to be COVID-19. However, all patients with a positive SARS-CoV-2 PCR test result also displayed residual immunological memory. Further, we could not collect samples for immunological analyses during the acute phase of infection due to quarantine regulations to protect other patients and staff. Therefore, flow cytometry data in most cases reflect the state several months after the acute infection. This also required that we used previously collected flow cytometry data to extrapolate the approximate level of B cell depletion at the time of acute infection. Importantly, however, such determinations are done in clinical routine before each RTX or OCR infusion, meaning that all included subjects on anti-CD20 had duration of B cell depletion determined at an individual level.

## STAR★Methods

### Key resources table

**Table:** 

REAGENT or RESOURCE	SOURCE	IDENTIFIER
**Antibodies**		

Mouse monoclonal anti-CD3	Biolegend	Cat# 344852; RRID:AB_2819985
Mouse monoclonal anti-CD19	Biolegend	Cat# 302269; RRID:AB_2860769
Mouse monoclonal anti-CD4	BD Horizon	Cat# 562971; RRID:AB_2744424
Mouse monoclonal anti-CD8	BD Pharmingen	Cat# 561026; RRID:AB_2033968
Mouse monoclonal anti-HLA-DR	Biolegend	Cat# 307657; RRID:AB_2572100
Mouse monoclonal anti-CD25	Biolegend	Cat# 302606; RRID:AB_314276
Mouse monoclonal anti-CD27	BD Horizon	Cat# 564894; RRID:AB_2739004
Mouse monoclonal anti-CD279 (PD-1)	Biolegend	Cat# 329939; RRID:AB_2563658
Mouse monoclonal anti-CD38	Biolegend	Cat# 303511; RRID:AB_493089
Mouse monoclonal anti-CD45RO	Biolegend	Cat# 304251; RRID:AB_2616917
Mouse monoclonal anti-CD185 (CXCR5)	Biolegend	Cat# 356919; RRID:AB_2562302
Mouse monoclonal anti-CD154	Biolegend	Cat# 310804; RRID:AB_314827
Mouse monoclonal anti-CD11a/CD18 (LFA-1)	Biolegend	Cat# 363410; RRID:AB_2716070
Rat monoclonal anti-CD197 (CCR7)	eBioscience	Cat# 46-1979-41; RRID:AB_10853035
Mouse monoclonal anti-CD127	BD Horizon	Cat# 566158; RRID:AB_2869742
Mouse monoclonal anti-IFN-γ	Biolegend	Cat# 502517; RRID:AB_493030
Mouse monoclonal anti-TNF-α	Biolegend	Cat# 502947; RRID:AB_2565857
Rat monoclonal anti-GM-CSF	BD Horizon	Cat# 562857; RRID:AB_2737843
Goat monoclonal anti- IgG Fc	Invitrogen	Cat# H10104; RRID:AB_2536546

**Biological Samples**		

Peripheral blood	This paper	Center for Neurology, Academic Specialist Center, Stockholm, Sweden

**Chemicals, Peptides, and Recombinant Proteins**		

PepTivator SARS-CoV-2 Prot_S	Miltenyi Biotec	Cat# 130-126-701
PepTivator SARS-CoV-2 Prot_S1	Miltenyi Biotec	Cat# 130-127-048
PepTivator SARS-CoV-2 Prot_N	Miltenyi Biotec	Cat# 130-126-699
PepTivator SARS-CoV-2 Prot_M	Miltenyi Biotec	Cat# 130-126-703
PepTivator EBV Consensus	Miltenyi Biotec	Cat# 130-099-764
PepTivator EBV EBNA-1	Miltenyi Biotec	Cat# 130-093-613

**Critical Commercial Assays**		

IFN-γ/IL-10/Granzyme B FluoroSpot plates	Mabtech	Cat# FSP-010710

**Software and Algorithms**		

Graphpad Prism 9.0.0	Graphpad	RRID:SCR_002798
FlowJo	Tree Star	https://www.flowjo.com/; RRID:SCR_008520

### Resource availability

#### Lead contact

Further information and requests for resources and reagents should be directed to and will be fulfilled by the Lead Contact, Faiez Al Nimer (faiez.al.nimer@ki.se).

#### Materials availability

This study did not generate new unique reagents.

#### Study design and subjects

The study design is shown in Figure 1. The core cohort comprised patients with a follow up at the Center of Neurology, Academic Specialist Clinic, Stockholm, Sweden and participating in the prospective COMBAT-MS study (COMparison Between All immunoTherapies for Multiple Sclerosis; Clinicaltrials.gov identifier: NCT03193866, EudraCT 2016-003587-39) and who were asked via post or during their regular appointment at the clinic to participate in this study. Six hundred twenty out of 932 COMBAT-MS participants at the center volunteered in this study. In addition, twelve additional pwMS enriched with history of COVID-19 and 15 healthy individuals were included. Because of the pandemic and restrictions on physical hospital visits, samples were collected between the end of July to the middle of November 2020.

All patients and controls were asked to fill in a questionnaire regarding history of possible COVID-19 symptoms or confirmed COVID-19 infection comprising the following questions; 1. Yes/No to a) episode of fever ≥ 38.5°C, b) cough, c) disturbed/loss of taste and/or smell d) durations of symptoms > 2 weeks; 2. Date of symptoms; 3. Yes/ No to a previously positive a) SARS-CoV-2 PCR test, b) SARS-CoV-2 antibody test. Furthermore, a blood test was sent for routine SARS-CoV-2 antibody detection (see below).

In a subcohort (n = 122) of patients enriched for positive symptoms according to the questionnaire and ongoing anti-CD20 therapy, we collected blood for serological (plasma) and cellular analyses. Peripheral blood mononuclear cells (PBMCs) were freshly isolated from sodium citrate-containing cell preparation tubes (BD Biosciences). All isolated PBMCs were cryopreserved in freezing media containing 10% dimethyl sulfoxide (DMSO; Sigma-Aldrich) and stored at -180°C. PBMCs from 8 samples from MS patients before the start of COVID-19 pandemic were also included in the analysis. Lastly, for the vaccination cohort, PBMCs from MS patients on anti-CD20 were collected before (n = 1), four (n = 10) and/or twelve (n = 4) weeks after SARS-CoV-2 mRNA vaccination. Seroconversion against the spike protein was tested at the hospital at the same date as the PBMCs sampling date and the results were later obtained from the patient’s journal. See Table S4 for cohort characteristics.

Study procedures were conducted under the following ethical permits approved by the Swedish ethical review authority; COMBAT-MS: 2017/32-31/4; STOPMSII: 2009/2107-31/2; 2020-00052, with written informed consent from participants.

### Method details

#### Serological SARS-CoV-2 analyses

Routine detection of SARS-CoV-2 IgG in serum was performed at Clinical Microbiology, Karolinska University Laboratory, Stockholm. Samples were analyzed by either the Elecsys® Anti-SARS-CoV-2 electro-chemiluminescence immunoassay in the Cobas 8000 system (Roche diagnostics) or YHLO’s SARS-CoV-2 IgG (Shenzhen Yhlo Biotech Co.) in the iFlash system. The data were collected in the clinical setting.

Since there is a considerable discussion about the sensitivity and specificity of the different assays in detecting anti-SARS-CoV-2 antibodies, we also included an additional method in the cohort of patients with more advanced immunological analyses. The protocol for detection of SARS-CoV-2 IgG using a multiplex bead array has been recently described (Rudberg et al., 2020). In brief, the assay measured IgG reactivity towards two different virus protein variants, the spike glycoprotein ectodomain produced in HEK cells (Spike S1S2 foldon), and the nucleocapsid protein C-terminal domain produced in *Escherichia coli* (Nucleocapsid C), using a multiplex antigen-bead array in a high throughput 384-well plates format using a FlexMap3D (Luminex Corp). Each antigen was coated on the surface of uniquely color-coded magnetic beads (bead ID) (Luminex Corp), and the antigen-reacting plasma IgG was captured and detected by fluorescent goat anti-hIgG (Invitrogen). Median fluorescent intensity (MFI) and bead-count were determined for each antigen (bead ID). As previously described, a cutoff of reactivity was defined for each antigen as the mean MFI + 6SD of 12 negative controls included in each analysis. Samples were regarded as positive when reactive to both viral antigens.

#### Data collection and anti-CD20 treatment

Patient baseline characteristics were extracted from the Swedish MS-registry (https://www.neuroreg.se/). For patients on anti-CD20 therapy, data included also number of infusions and date of latest infusion before COVID-19-like symptoms. Three patients received rituximab between COVID-19-like symptoms and blood sampling and the date of rituximab treatment was noted. In addition, data were collected at the time points available for the number of T (CD3^+^) and B (CD19^+^) cells for patients on anti-CD20 treatment. These analyses were conducted in clinical routine and performed before each new infusion at the Department of Clinical Immunology, Karolinska University Hospital, Sweden. The dates of last rituximab infusion in relation to onset of symptoms, together with flow cytometric data of B cells in previous treatment cycles were used to estimate the B cell status at symptom onset. According to this, patients were categorized as having; a) complete B cell depletion (<0.01; B cell count x 10^9^ / L), b) partial B cell repletion (0.01-0.08; B cell count x 10^9^ / L) and c) complete B cell repletion (>0.08; B cell count x 10^9^ / L).

Total lymphocyte counts were also extracted for patients on other treatments testing positive for SARS-CoV-2 antibodies or T-cell IFN-γ SARS-CoV-2 reactivity.

#### B- and T- cell characterization of PBMCs

PBMCs were thawed in complete RPMI (cRPMI) medium (RPMI 1640 (R8758, Sigma Aldrich) supplemented with 10% heat inactivated fetal bovine serum (F7524, Sigma Aldrich), 100 U/mL penicillin and 100 μg/mL streptomycin (P4458, Sigma Aldrich)), washed and stained for surface markers using the fluorochrome-conjugated antibodies in the presence of Live Dead (Invitrogen) for 15-30 min at RT (Key Resources Table; Figure S5A). For intracellular staining, cells were fixed and permeabilized using Cytofix/Cytoperm kit (BD) according to manufacturer’s instructions. Measurements were performed on an Aurora spectral cytometer (Cytek), and data were analyzed with FlowJo v.10 (Tree Star).

#### FluoroSpot

Pre-coated human IFN-γ/IL-10/Granzyme B FluoroSpot plates (FSP-010710, Mabtech) were washed three times with sterile PBS (Sigma-Aldrich) and blocked for 30 min with cRPMI. For the peptide stimulation, overlapping peptides spanning the SARS-CoV-2 nucleocapsid phosphoprotein (Prot_N), membrane glycoprotein (Prot_M) and spike glycoprotein (Prot_S, Prot_S1) (PepTivator®, Miltenyi Biotec) were added to the respective wells in a final concentration of 1 μg/mL each. Anti-CD3 was used as a positive control in accordance to the manufacturer’s protocol. Wells lacking both anti-CD3 and peptides were used as negative control. PBMCs were thawed in 37°C water bath and washed twice with cRPMI. The cell count and viability was measured by using a Luna-II automated cell counter (Logos Biosystems). For the positive control, 1.25 x 10^5^ PBMCs and for all other conditions 2.5 x 10^5^ PBMCs were seeded per well. Every sample and condition was tested in duplicate. The plates were incubated for 44 h in a 37°C humidified incubator with 5% CO_2_. Plates were developed under non-sterile conditions according to the manufacturer’s protocol and read in an automated FluoroSpot-reader (IRIS, Mabtech). Data are presented as delta-spot forming units (ΔSFUs) calculated as the mean spot forming units (SFUs) for each condition duplicate minus the mean SFUs from the respective negative controls. Samples with high IFN-γ background (> 50 SFUs) or low IFN-γ anti-CD3 response (< 200 SFUs) were excluded from further analysis (in total 5 pwMS; 1 rituximab, 1 natalizumab, 1 dimethyl fumarate and 2 pre-COVID-19 baseline samples). Neither interleukin 10 (IL-10) nor Granzyme B (GrB) secretion in FluoroSpot was specific for SARS-CoV-2 and was excluded from further analyses (Figures S5B and S5C). Of note, samples taken within a few days after resolution of COVID-19-like symptoms displayed higher numbers of SFUs without antigen stimulation, suggesting an activated immune state close to an infection (Figure S5D).

#### Cell stimulation, extra- and intracellular cytokine staining

PBMCs were thawed as previously described and rested overnight at 37°C with 5% CO_2_. For each stimulation and control, 1 x 10^6^ cells were seeded in a total volume of 200 μL as single samples in a U-bottom 96-well plate. Anti-CD3 was used as positive control and prepared as previously described. As an additional control, EBV Consensus and EBNA-1 peptides were pooled together (0.5 μg/mL each, Miltenyi Biotec). Wells containing only medium were used as negative control. For the SARS-CoV-2 peptide stimulation, the peptides were divided into two pools, one containing all four proteins; Prot_N, Prot_M, Prot_S and Prot_S1 and one containing only Prot_N and Prot_S1 (each at 1 μg/mL). Monensin (0.6 μL) and brefeldin A (1.0 μL) (BD Bioscience) were added to each well before seeding the cells. The plate was incubated for 4 h in a 37°C humidified incubator with 5% CO_2_. LFA-1 staining was performed as previously described (Dimitrov et al., 2018; Schollhorn et al., 2021), by adding LFA-1 m24 Ab to the culture and cells incubated for 15 min at room temperature. Then EDTA was added for an additional 10 min RT. Cells were washed, fixed and permeabilized as described above and thus antibody mix was added (Key Resources Table; Figure S5A). Measurements were performed on an Aurora spectral cytometer (Cytek), and data were analyzed with FlowJo v.10 (Tree Star).

#### Statistical analysis

Statistical analyses were performed with GraphPad Prism software version 9.0.0. Group analyses were tested with a two-sided Mann-Whitney test or Kruskal Wallis with Dunn's post-hoc multiple comparison test. Correlation was calculated using Spearman's rank correlation test. *P* values are reported in the figure and figure legend when relevant.

## Data Availability

This study did not generate/analyze [datasets/code].

## Supplemental information

Document S1. Tables S2–S4 and Figures S1–S5

Table S1. Detailed characteristics of the study cohort, related to Table 1
