# Supplementary material for: Development of humoral and cellular immunological memory against SARS-CoV-2 despite B cell depleting treatment in multiple sclerosis
Source: iScience. 2021 Sep 2;24(9):103078. doi: 10.1016/j.isci.2021.103078 (PMC8410640; doi:10.1016/j.isci.2021.103078)
Supplement: Document S1. Tables S2–S4 and Figures S1–S5 [file mmc1.pdf]

iScience, Volume *24*

Supplemental information

Development of humoral and cellular immunological memory against SARS-CoV-2 despite B cell depleting treatment in multiple sclerosis

Klara Asplund Högelin, Nicolas Ruffin, Elisa Pin, Anna Månberg, Sophia Hober, Guro Gafvelin, Hans Grönlund, Peter Nilsson, Mohsen Khademi, Tomas Olsson, Fredrik Piehl, and Faiez Al Nimer

Table S2. Maximum number of individuals assessed for each assay, Related to STAR Methods, Figure 1 and Table 1.

Participants and treatment	n	ECLIA	Multiplex bead array	*Both ECLIA and Multiplex bead array*	FluoroSpot	*Excluded from FluoroSpot analysis*	Flow cytometry with peptide stimulation
**HC**	15	9	10	*4*	15	*0*	9
**Interferon-beta**	1	1	0	*0*	1	*0*	0
**Teriflunomide**	1	1	1	*1*	1	*0*	0
**Dimethyl fumarate**	11	11	11	*11*	11	*1*	4
**Fingolimod**	7	6	7	*6*	5	*0*	0
**Cladribine**	3	3	2	*2*	3	*0*	1
**Natalizumab**	19	19	17	*17*	19	*1*	3
**Rituximab**	71	71	68	*68*	71	*1*	19
**Ocrelizumab/ Ofatumumab**	5	4	5	*4*	5	*0*	0
**HSCT**	4	4	4	*4*	4	*0*	0

ECLIA = electro-chemiluminescence immunoassay; HSCT = hematopoietic stem cell transplantation.

Table S3. Characteristics of the rituximab study population, Related to Figure 5 and Supplementary Figure 3.

Rituximab Depletion status	n	Age in years mean ± SD	Female No (%)	EDSS median (min; max)	Patients with COVID-19-like symptoms No (%)	Days from symptoms median (min; max)	Antibody ECLIA and/or multiplex bead array Positive/ Negative No	T-cell reactivity Positive/ Negative No
**All pwMS with rituximab**	70	43.9 ± 10.1	54 (77%)	2 (0; 6)	62 (89%)	162 (43; 245)	16; 54	24; 46
**Repletion**	9	43.8 ± 10.3	8 (89%)	1.5 (0; 3)	9 (100%)	131 (43; 197)	3; 6	3; 6
**Partial repletion**	19	41.8 ± 11.4	14 (74%)	2 (0; 5)	19 (100%)	145 (45; 236)	5; 14 *	7; 12
**Depletion**	32	45.1 ± 9.3	25 (84%)	2.5 (0; 6)	32 (100%)	174 (43; 245)	7; 25 *	12; 20
**No symptoms or N/A**	10	43.9 ± 11.1	7 (70%)	2 (0; 4)	2 (20%)	N/A	1; 9	2; 8
**All COVID19^{+} pwMS with rituximab**	26	42.1 ± 9.8	17 (65%)	2 (0; 3.5)	24 (92%)	143 (45; 245)	16; 10	24; 2
**Repletion**	3	42.7 ± 11.0	2 (66%)	1.5 (1; 2)	3 (100%)	105 (101; 131)	3; 0	3; 0
**Partial repletion**	7	34.1 ± 8.5	5 (71%)	2 (0; 3)	7 (100%)	132 (45; 234)	5; 2 *	7; 0
**Depletion**	14	46.6 ± 8.6	9 (64%)	2.25 (0; 3.5)	14 (100%)	171 (63; 245)	7; 7 *	12; 2
**No symptoms or N/A**	2	38.0 ± 4.2	1 (50%)	1 (0; 2)	0 (0%)	N/A	1; 1	2; 0
**All Ab^{+} COVID-19^{+} with rituximab**	16	40.8 ± 9.7	9 (56%)	2 (0; 3.5)	15 (94%)	136 (45; 245)	16; 0 *	14; 2
**All Ab^{-} COVID-19^{+} with rituximab**	10	44.2 ± 10.1	8 (80%)	2.25 (0; 3.5)	9 (90%)	181 (63; 234)	0; 10*	10; 0

Ab = antibody; ECLIA = electro-chemiluminescence immunoassay; EDSS = expanded disability status scale; N/A = Not applicable; pwMS = persons with multiple sclerosis.

*One pwMS not tested with multiplex bead array.

Table S4. Characteristics of the vaccination study population, Related to Figure 6.

	Study participants	n	Age in years mean ± SD	Female No (%)	EDSS median (min; max)
SARS-CoV-2 vaccination cohort	MS patients	11	44.0 ± 7.0	8 (73%)	1.5 (0; 7)
	Ocrelizumab	1	36	1 (100%)	2 (2; 2)
	Ofatumumab	2	49.0 ± 4.2	2 (100%)	0.5 (0; 1)
	Rituximab	8	43.8 ± 7.2	5 (63%)	1.75 (0; 7)

EDSS = expanded disability status scale.

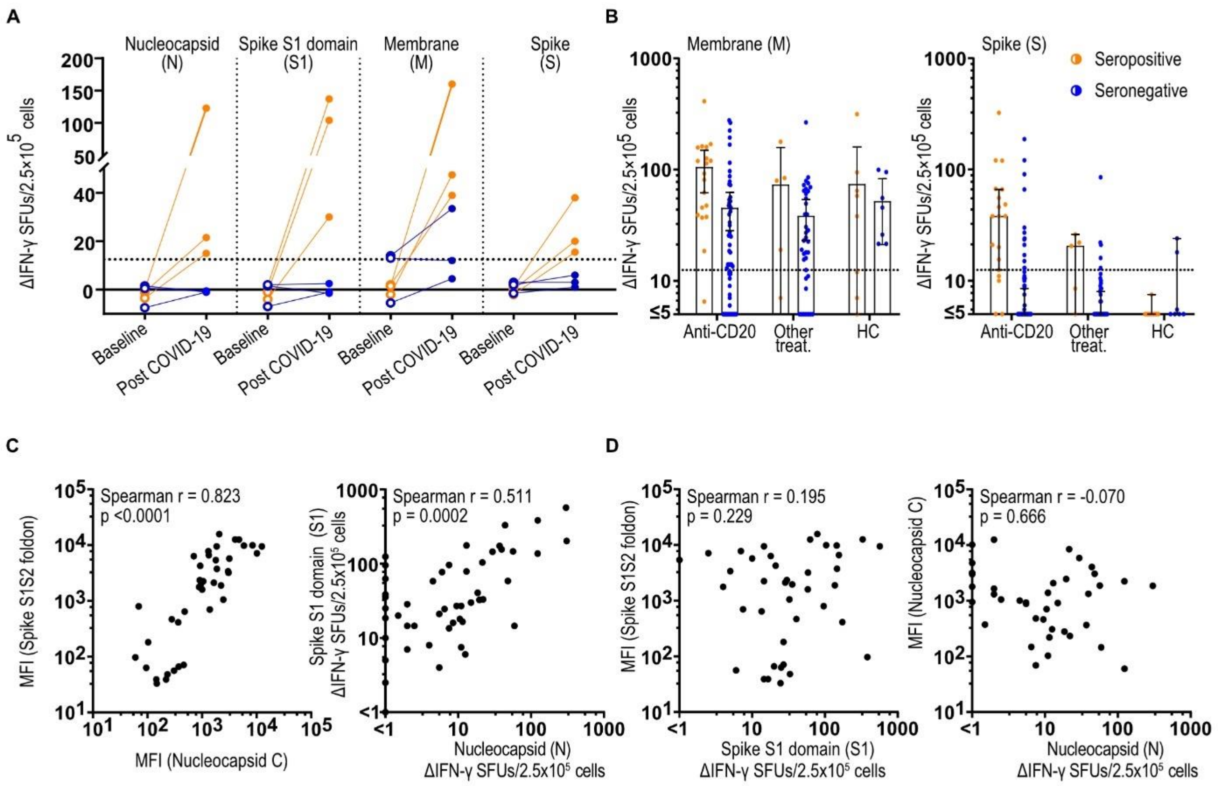

Figure S1. Specificity of the four protein domain peptides for SARS-CoV-2 and correlations between humoral and T-cell reactivity for the spike and nucleocapsid domain, Related to STAR Methods and Figure 2.

(**A**) Number of ΔIFN-γ SFUs for the nucleocapsid (N), spike 1 (S1), membrane (M) and spike (S) domain from 3 seropositive and 3 seronegative patients (n=6) after the COVID-19 pandemic and as compared to samples from the same patients before the pandemic. (**B**) Number of ΔIFN-γ SFUs after stimulation with M (n=132) or S peptides (n=132) in seropositive or seronegative patients on anti-CD20 (n=75) or other immunosuppressive treatment (n=42) and in HC (n=15). (**C**) Correlation of SARS-CoV-2 spike with nucleocapsid domain antibody levels (n=40) and SARS-CoV-2 spike with nucleocapsid domain ΔIFN-γ SFUs (n=49). (**D**) Correlation of MFI with ΔIFN-γ SFUs measured for the SARS-CoV-2 spike (n=40) and nucleocapsid domain (n=40).

Spearman r and p value are shown. Dots represent individual data points. Box plots represent median and 95% CI.

HC = healthy controls; MFI = median fluorescent intensity; SFU = spot forming unit.

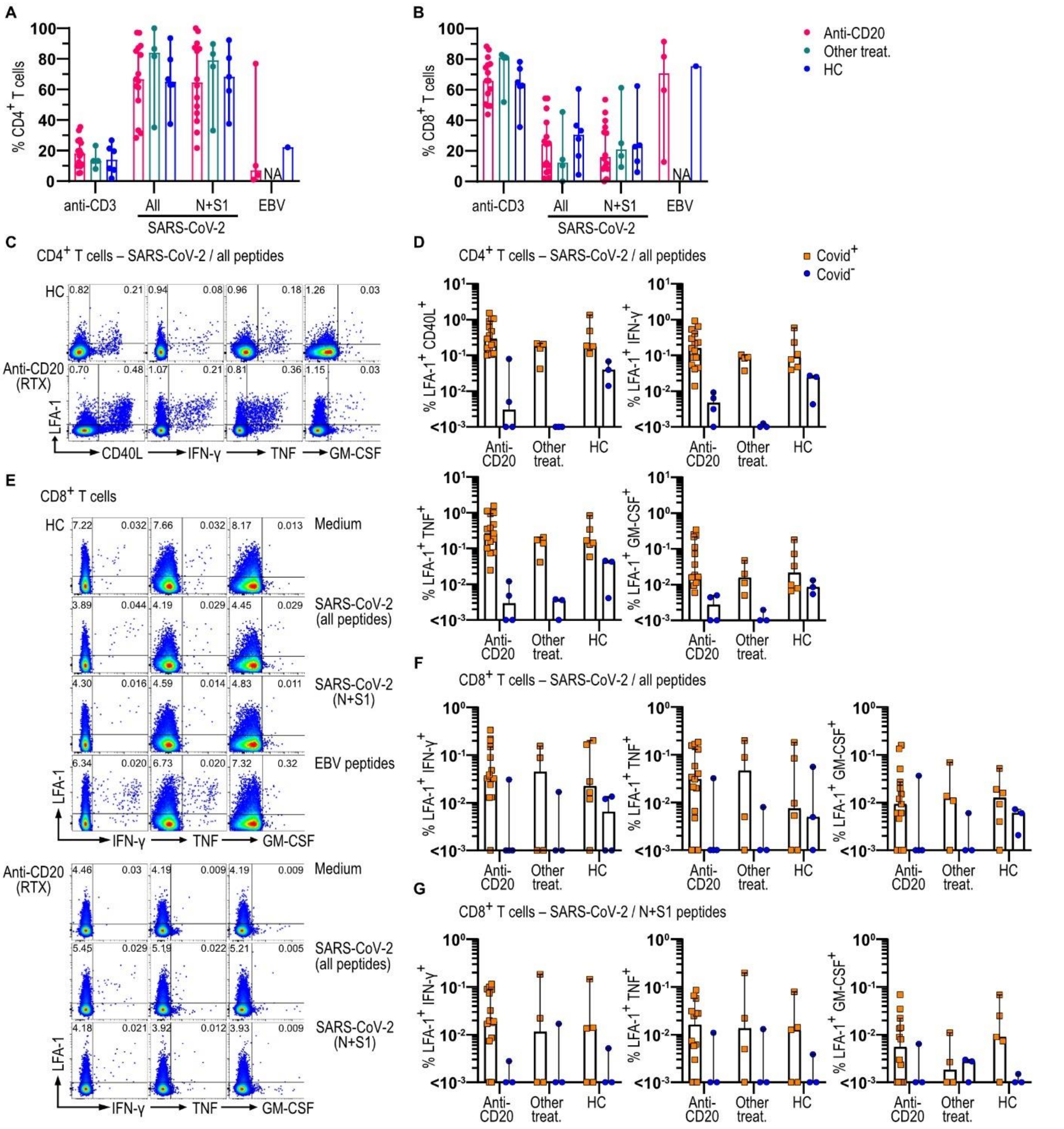

Figure S2. SARS-CoV-2 specific T-cell from MS patients are functional, Related to STAR Methods and Figure 3.

(**A-B**) Percentages of $CD4^+$ and $CD8^+$ T-cells among $LFA1^+$ IFN-γ^+ T-cells following anti-CD3, SARS-CoV-2 or EBV peptide stimulation of PBMCs from Covid$^+$ pwMS with anti-CD20 (n=15) or other therapies (n=4) and HC (n=6). (**C**) Representative dotplots of $CD4^+$ T-cell expression of LFA-1 with CD40L, IFN-γ, TNF and GM-CSF from one HC and one pwMS with anti-CD20 treatment as in Figure 3F following a culture with all four SARS-CoV-2 peptides N, S, S1 and M. (**D**) Percentages of LFA-$1^+$$CD40L^+$, LFA-$1^+$ IFN-γ^+, LFA-$1^+$$TNF^+$ and LFA-1^+GM-CSF^+ among $CD4^+$ T-cells in HC (n=9) and pwMS with anti-CD20 therapy (n=18) or with other therapies (n=7) as in (C) and Figure 3F. (**E**) Representative dotplots of $CD8^+$ T-cell expression of LFA-1 with CD40L, IFN-γ, TNF and GM-CSF from one HC and one pwMS with anti-CD20 treatment as in Figure 3F following a culture with medium, all four SARS-CoV-2 peptides (N, S, S1 and M), peptides N and S1 only, or EBV peptides. (**F-G**) Percentages of LFA-$1^+$$CD40L^+$, LFA-$1^+$ IFN-γ^+, LFA-1^+ TNF^+ and LFA-1^+GM-CSF^+ among $CD8^+$ T-cells in HC (n=9) and pwMS with anti-CD20 therapy (n=18) or with other therapies (n=7) as in (E) following culture with all four SARS-CoV-2 peptides (N, S, S1 and M) or SARS-CoV-2 peptides N and S1 only.

Dots represent individual data points. Box plots represent median and 95% CI. For (D), (F) and (G), background values (medium) were subtracted.

EBV = Epstein-Barr virus; HC = healthy controls; MFI = median fluorescent intensity; PBMC = peripheral blood mononuclear cells; pwMS = persons with multiple sclerosis; RTX = rituximab.

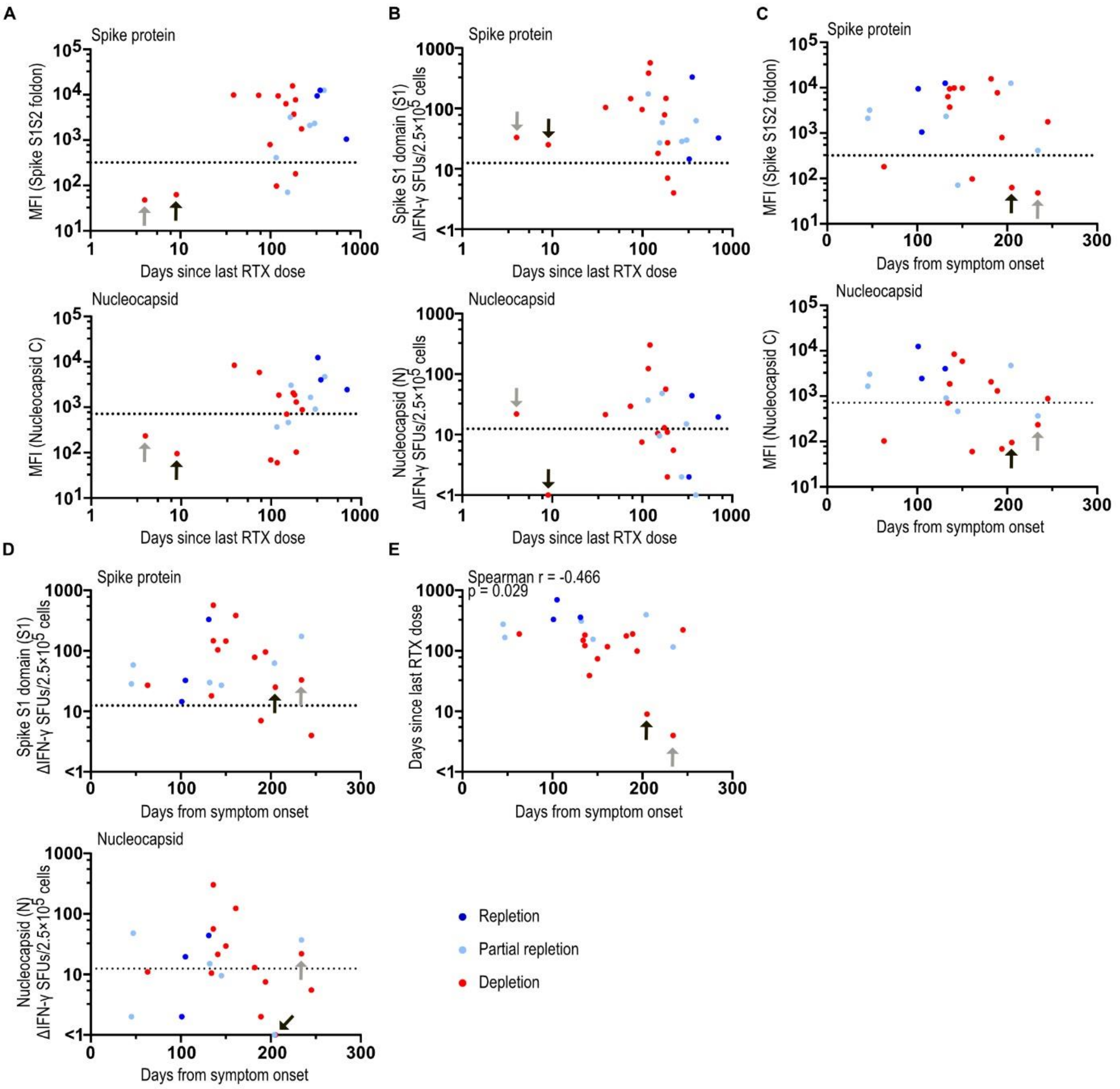

Figure S3. Humoral and cellular immunological memory to SARS-CoV-2 compared to time since last RTX dose and time from symptom onset, Related to Figure 5 and Table S3.
(**A-B**) Correlation of SARS-CoV-2 antibody levels as measured by MFI of S1S2 or N or ΔIFN-γ SFUs after stimulation with S1 or N peptides with time (days) since last RTX dose. Relation to B-cell depletion status; n=3 repletion, n=6 partial repletion, n=13 depletion. (**C-D**) Correlation of SARS-CoV-2 antibody levels of S1S2 or N or ΔIFN-γ SFUs after stimulation with S1 or N peptides respectively with time (days) from symptoms to sampling. Relation to B-cell depletion status; n=3 repletion, n=6 partial repletion, n=13 depletion. (**E**) Correlation between days since last RTX dose and days from symptom onset to sampling. Relation to B-cell depletion status; n=3 repletion, n=6 partial repletion, n=13 depletion.
Spearman r and p value are shown. Dots represent individual data points. Box plots represent median and 95% CI. The black and grey arrow indicate two patients that were seronegative but displayed T-cell reactivity to SARS-CoV-2 in all figures respectively.
MFI = median fluorescent intensity; RTX = rituximab; SFU = spot forming unit.

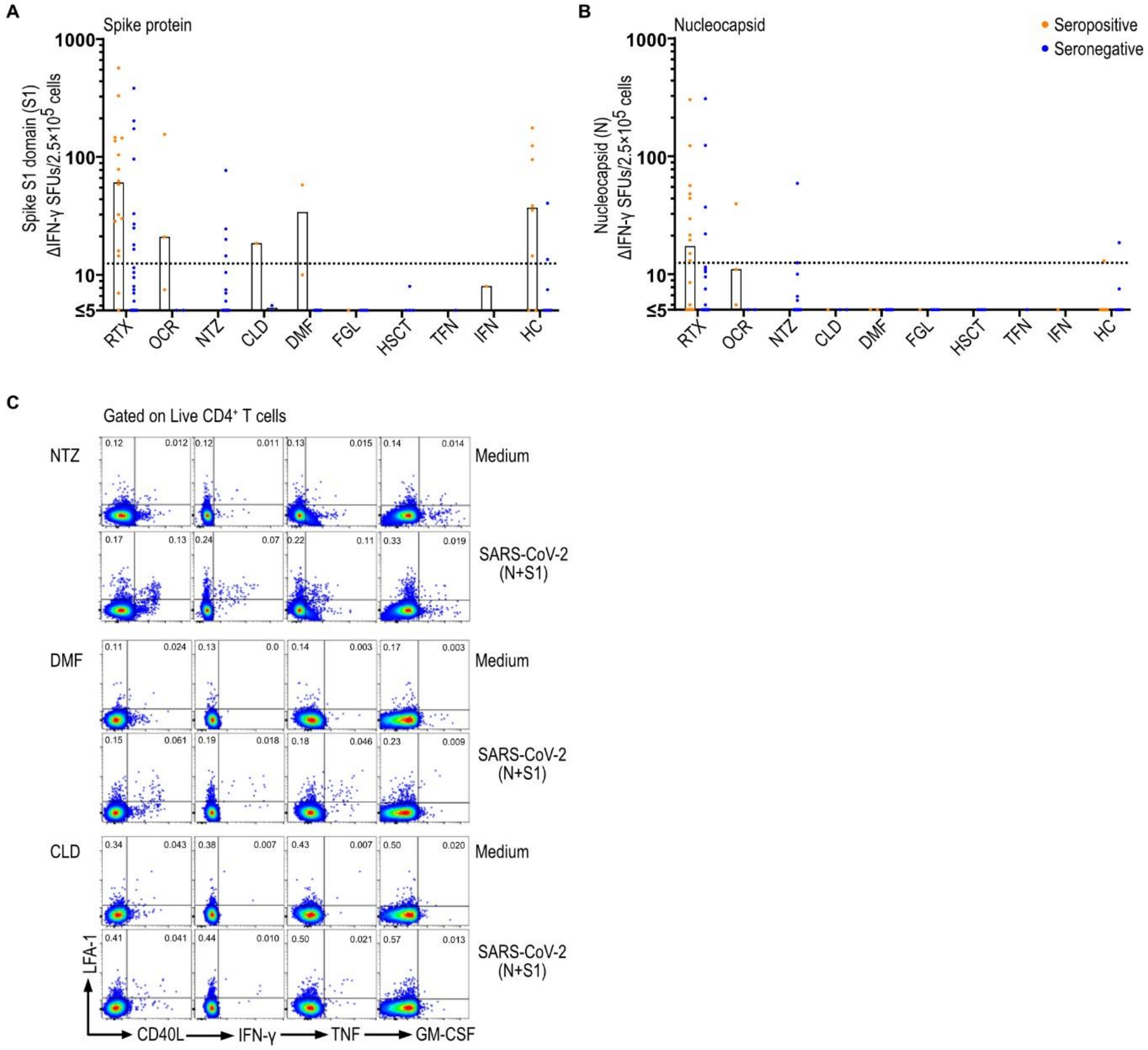

Figure S4. Immunological memory and functionality of cellular immunological memory in pwMS on other immunomodulatory treatments *, Related to STAR Methods.
(**A**) Number of ΔIFN-γ spot-forming units measured by FluoroSpot after stimulation with S1 (n=132) or N peptides (n=132) in pwMS on RTX (seropositive, n=16; seronegative n=54), OCR (seropositive, n=3; seronegative n=2), NTZ (seropositive, n=0; seronegative n=18), CLD (seropositive, n=1; seronegative n=2), DMF (seropositive, n=2; seronegative n=8), FGL (seropositive, n=1; seronegative n=4), HSCT (seropositive, n=0; seronegative n=4), TFN (seropositive, n=0; seronegative n=1), IFN (seropositive, n=1; seronegative n=0) or HC (seropositive, n=8; seronegative n=7). Dots represent individual data points. (**B**) Dotplots of $CD4^+$ T-cells expression of LFA-1 with CD40L, IFN-γ, TNF and GM-CSF from one pwMS on NTZ, one pwMS on DMF and one pwMS on CLD following a culture with medium or in the presence of SARS-CoV-2 peptides N and S1.

*****Of note, four patients on NTZ treatment that were seronegative displayed ΔIFN-γ SFU ≥ 12.5 ΔSFU/ $2.5x10^5$ cells. In addition, the two seropositive pwMS on DMF and two seropositive pwMS on FGL displayed lymphopenia grade 2 and grade 3, respectively, when tested in blood during the last year. Due to grade 3 lymphopenia, too few cells were collected from one FGL pwMS and thus could not be tested in T-cell assays. Interestingly, a pwMS on CLD treatment had to wait for the second-year treatment cycle for 5.5 months before the absolute lymphocytes count increased to 800/ µL. The patient reported a negative SARS-CoV-2 antibody test done in another laboratory just before the second year treatment cycle and one month after the last cladribine dose he tested positive for SARS-CoV-2 antibodies while also displaying T-cell immunological memory with lymphocyte number 300/ µL.

CLD = cladribine; DMF = dimethyl fumarate; FGL = fingolimod; HC = healthy controls; HSCT = hematopoietic stem cell transplantation; IFN = interferon-beta; NTZ = natalizumab; OCR = ocrelizumab; pwMS = persons with multiple sclerosis; RTX = rituximab; SFU = spot forming unit; TFN = teriflunomide.

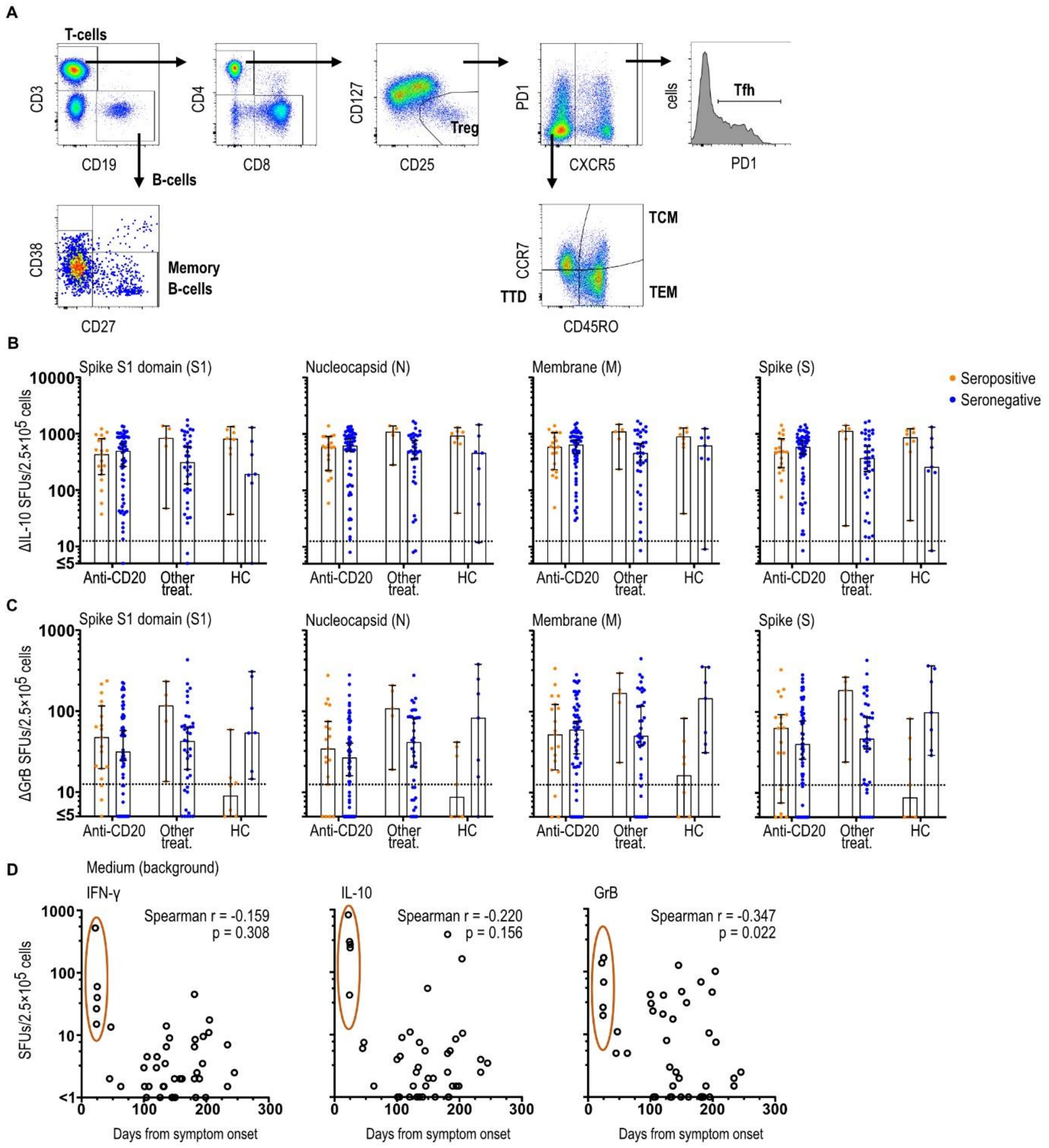

Figure S5. Flow cytometry gating strategy and Interleukin-10 and Granzyme B as measured in FluoroSpot, Related to STAR Methods.

(**A**) Exemplar gating strategy for flow cytometry staining for extracellular B-/T- cell phenotyping from a representative donor sample. (**B**) Number of ΔIL-10 SFUs measured by FluoroSpot for the nucleocapsid (N), spike 1 (S1), membrane (M) and spike (S) domain from patients on anti-CD20 treatment (seropositive, n=19; seronegative n=56), other immunosuppressive treatment (seropositive, n=5; seronegative n=37) or in HC (seropositive, n=8; seronegative, n=7). (**C**) Number of ΔGrB SFUs measured by FluoroSpot for the nucleocapsid (N), spike 1 (S1), membrane (M) and spike (S) domain from patients on anti-CD20 treatment (seropositive, n=19; seronegative n=56), other immunosuppressive treatment (seropositive, n=5; seronegative n=37) or in HC (seropositive, n=8; seronegative, n=7). (**D**) Distribution of the background T-cell response as measured by IFN-γ, IL-10 and GrB SFUs and relation to days from symptoms until sampling (n=43).

Box plots represent median and 95% CI.

GrB = granzyme B; HC = healthy controls; IL-10 = interleukin 10; SFU = spot forming unit; TCM cells = central memory T-cells; TEM cells = effector memory T-cells; Tfh = T follicular helper cells; Treg = T regulatory; TTD cells = terminally differentiated T-cells.
